# Sample size requirements are not being considered in studies developing prediction models for binary outcomes: a systematic review

**DOI:** 10.1186/s12874-023-02008-1

**Published:** 2023-08-19

**Authors:** Paula Dhiman, Jie Ma, Cathy Qi, Garrett Bullock, Jamie C Sergeant, Richard D Riley, Gary S Collins

**Affiliations:** 1 Centre for Statistics in Medicine, Nuffield Department of Orthopaedics, Rheumatology and Musculoskeletal Sciences, University of Oxford, Oxford, OX3 7LD UK; 2 Population Data Science, Faculty of Medicine, Health and Life Science, Swansea University Medical School, Swansea University, Singleton Park, Swansea, SA2 8PP UK; 3 Department of Orthopaedic Surgery, Wake Forest School of Medicine, Winston-Salem, NC USA; 4 Centre for Sport, Exercise and Osteoarthritis Research Versus Arthritis, University of Oxford, Oxford, UK; 5 Centre for Biostatistics, University of Manchester, Manchester Academic Health Science Centre, Manchester, M13 9PL UK; 6 Centre for Epidemiology Versus Arthritis, Centre for Musculoskeletal Research, University of Manchester, Manchester Academic Health Science Centre, Manchester, M13 9PT UK; 7 Institute of Applied Health Research, College of Medical and Dental Sciences, University of Birmingham, B15 2TT Birmingham, UK

**Keywords:** Sample size, Methodology, Prediction model

## Abstract

**Background:**

Having an appropriate sample size is important when developing a clinical prediction model. We aimed to review how sample size is considered in studies developing a prediction model for a binary outcome.

**Methods:**

We searched PubMed for studies published between 01/07/2020 and 30/07/2020 and reviewed the sample size calculations used to develop the prediction models. Using the available information, we calculated the minimum sample size that would be needed to estimate overall risk and minimise overfitting in each study and summarised the difference between the calculated and used sample size.

**Results:**

A total of 119 studies were included, of which nine studies provided sample size justification (8%). The recommended minimum sample size could be calculated for 94 studies: 73% (95% CI: 63–82%) used sample sizes lower than required to estimate overall risk and minimise overfitting including 26% studies that used sample sizes lower than required to estimate overall risk only. A similar number of studies did not meet the ≥ 10EPV criteria (75%, 95% CI: 66–84%). The median deficit of the number of events used to develop a model was 75 [IQR: 234 lower to 7 higher]) which reduced to 63 if the total available data (before any data splitting) was used [IQR:225 lower to 7 higher]. Studies that met the minimum required sample size had a median c-statistic of 0.84 (IQR:0.80 to 0.9) and studies where the minimum sample size was not met had a median c-statistic of 0.83 (IQR: 0.75 to 0.9). Studies that met the ≥ 10 EPP criteria had a median c-statistic of 0.80 (IQR: 0.73 to 0.84).

**Conclusions:**

Prediction models are often developed with no sample size calculation, as a consequence many are too small to precisely estimate the overall risk. We encourage researchers to justify, perform and report sample size calculations when developing a prediction model.

**Supplementary Information:**

The online version contains supplementary material available at 10.1186/s12874-023-02008-1.

## Introduction

Sample size is a crucial design consideration for any research study. It has a key role when developing and validating a prediction model as emphasised in the TRIPOD reporting guideline (‘Item 8: Explain how the study size was arrived at’) and the PROBAST risk of bias assessment tool for prediction model studies (Signalling question 4.1 ‘Were there a reasonable number of participants with the outcome?’) [1, 2]. Using a sample size that is too small when developing a prediction model leads to imprecise parameter estimates and increases the risk of overfitting, which can yield inaccurate and unstable predictions leading to poor model performance when evaluated in ‘new’ individuals from the same population, and ultimately limits generalisability of the model [3].

Increased risk of bias can generally be introduced through four domains: participants, outcome, predictors and analysis. Sample size informs the assessment of the risk of bias in the analysis domain and captures the risk of overfitting in the developed model, finding potentially spurious associations and imprecise predictor parameter estimation, which will impact of the predictions from the model. Reviews have found that inadequate sample sizes are a key contributor to high risk of bias in prediction model studies [4, 5]. For example, Wynants et al. found that 67% of studies were at high risk of bias due to inadequate sample sizes [4].

Researchers might think they can ‘overcome’ the limitation of using a too small sample when developing a prediction model by using penalisation and shrinkage methods (e.g., Least Absolute Shrinkage and Selection Operator (LASSO) regression, Ridge regression and elastic net). However, these methods do not solve the problem, since the shrinkage parameters are estimated with uncertainty when sample size is small, leading to unreliable prediction models [6, 7].

Therefore, sample size requirements should be considered, defined and justified when planning and designing a prediction model development study, and should be reported in the protocol and the final report, with enough details to allow replication. The rule of thumb of 10 outcome events per variable (EPV) have typically been used to guide the calculation and justification of the sample size for developing a prediction model [4, 8–10]. However, this rule of thumb has been shown to have no rationale, especially in prediction model research, as its evidence base is mainly informed by simulation studies that investigate the performance of estimating covariate-outcome relationships. The 10 EPV strategy also disregards the progression in methodological guidance for prediction model research and is thus widely cautioned against [11].

In 2019, Riley et al. published a series of papers providing guidance to help researchers calculate the minimum sample size requirements for their study, when developing models predicting binary, time-to-event or continuous outcomes [12, 13]. For a model predicting a binary outcome, the sample size calculation is based on the number of candidate predictor parameters for the model, the outcome proportion expected in the development data set, and the anticipated Cox Snell R^2^ (which can also be approximated from the anticipated c-statistic) [13]. The sample size calculation derives the minimum sample size to satisfy three criteria: (1) small overfitting defined by an expected shrinkage of predictor effects (reduction or penalty of predictor parameter estimates) by 10% or less, (2) small absolute difference of 0.05 in the model’s apparent and adjusted Nagelkerke’s R^2^ value, (3) precise estimation (within +/- 0.05) of the average outcome risk in the population [14]. The sample size formulae are implemented using the *pmsampsize* module in R and Stata, to enhance accessibility and ease of use [15]. However, it is unknown whether this approach is adhered to in practice, or if 10 EPV or other approaches are prevalent.

The aim of this article is to systematically review the sample size used in studies developing a prediction model for a binary outcome using logistic regression. We examine if and how sample size calculations are used or justified. We also calculate, using information reported in the included studies, the Riley et al. minimum sample size required for each study [13], and compare this with the sample size based on the 10 EPV rule of thumb approach and the actual sample size used. We then conclude with some recommendations.

## Methods

We conducted a systematic review of prediction models developed using logistic regression. We registered the study and uploaded the study protocol and data extraction form on the Open Science Framework (osf.io/qydmk). The study is reported following the Preferred Reporting Items for Systematic Reviews and Meta-Analyses (PRISMA) statement and PRISMA-S (an extension to the PRISMA Statement for Reporting Literature Searches in Systematic Reviews) [16, 17].

### Eligibility criteria

Primary studies developing a clinical prediction model for a binary, patient-related health outcome using logistic regression (penalised or unpenalised), published between 01 and 2020 and 31 July 2020 were included. We did not restrict the search to any specific clinical specialty or study design (e.g., randomised trials, cohort studies, case-control studies, registry-based studies).

Studies were excluded if they were:

- developing models using any other method than logistic regression.
- predicting time-to-event outcomes.
- imaging, risk or prognostic factor only studies (where the aim is not to develop prediction model, rather assess the association of particular risk or prognostic factor(s) with a particular outcome), studies that only externally evaluate existing models on new data (validation only studies), and imaging, genetic and molecular studies (that reflect prediction modelling in higher dimensional settings).
- conference abstracts.
- studies where the full-text could not be retrieved.

Studies were restricted to those published in the English language and involved humans.

### Information sources and search strategy

We searched for eligible studies using the PubMed (via www.pubmed.ncbi.nlm.nih.gov) medical literature database published between 01 and 2020 and 31 July 2020 (the search strategy was run on 3 August 2020).

The search strategy included a combination of general prediction (e.g., ‘prediction’, ‘prognosis’), model (e.g., ‘logistic’, ‘model’, and ‘regression’), and model performance (e.g., ‘discrimination’, ‘calibration’, and ‘area under the curve’) search terms. Publications satisfying the three strands of the search were then restricted to studies published within the search dates. The full search strategy is provided in Supplementary Box 1.

### Study selection, data extraction and data management

Publication records from PubMed were imported into Endnote reference software where they were de-duplicated [18]. Publications were then imported into Rayyan web application where any remaining duplicates were removed, and the titles and abstracts of the remaining publications were screened for inclusion [19].

Two independent reviewers screened the titles and abstracts and then the full texts of the publications using the defined eligibility criteria (PD and JM). All included articles were then allocated to two independent researchers from a combination of four reviewers (PD, JM, GB, CQ) for a double data extraction using a standardized data extraction form. Disagreements in data extraction were discussed and resolved between the two reviewers, and adjudicated by an additional reviewer (GSC), if necessary.

Data from the included publications were extracted using data extraction forms, developed using items specific to sample size from the CHARMS and TRIPOD checklists [1, 20]. We included additional items required for calculating the minimum sample size using formal sample size calculations by Riley et al. [13]. The data extraction form was piloted on five papers and amended as necessary. In studies developing more than one model, data was extracted on the first model mentioned in the paper. If multiple models were developed for multiple outcomes, we extracted on the model for the primary outcome only.

### Data items

For each included study, we extracted descriptive data on the overall publication, including clinical specialty, study design, target population and outcome to be predicted. We extracted the information on the number of candidate predictors and the number of candidate predictor parameters (i.e., each potential regression coefficients in the prediction model equation, for example, a categorial predictor with three groups will have two regression coefficients that need to be estimated). If the number of candidate predictor parameters was not reported or could not be calculated, we assumed it to be the number of candidate predictors. We extracted the sample size and number of events used for developing the prediction model (to calculate the outcome rate). We also extracted the Cox-Snell R^2^ value (or the c-statistic if the R^2^ value was not reported), which are needed to calculate the minimum sample size requirements for the study using formulae from Riley et al. [13].

We extracted information on methods, including the presence of sample size calculation or justification, and internal validation type (e.g., random split sample, bootstrapping). We also extracted information from the results, including the total available sample size and number of events (*before* any potential discarding or data splitting), the sample size (and number of events) used for developing the prediction model (*after* any potential discarding or data splitting), and the calibration slope and calibration-in-the-large from the developed model.

The primary outcome was the difference between the sample size used to develop the prediction model *after* any potential discarding or data splitting and the minimum sample size requirement as calculated by Riley et al. formulae (done separately for the minimum sample size to meet all three criteria and the minimum sample size to precisely estimate the overall risk, namely criterion 3). We specifically examined criterion 3, separately to criteria 1 and 2, to isolate the sample size required to precisely estimate overall risk and because this criteria can be considered the absolute lowest sample size that could be accepted when developing a prediction model. The secondary outcome was the difference between the total sample size available to develop the model *before* any potential discarding or data splitting and the minimum sample size requirement as calculated by Riley et al. formulae (done separately for the minimum sample size to meet all three criteria and minimum sample size to precisely estimate the overall risk, criterion 3). Risk of bias of individual studies was not assessed.

### Data analysis

Data were summarised using descriptive statistics and visual plots and a narrative synthesis was used to describe reporting detail. For each study, we calculated the event per predictor parameter (EPP) using:

$$\begin{gathered} EPP = number\,of\,events/ \hfill \\\,\,\,\,\,\,\,\,\,\,\,\,\,\,\,\,\,\,\,\,\,\,number\,of\,candidate\,predictor\,parameters \hfill \\ \end{gathered}$$

We used the Riley et al. [13] formulae to calculate the sample size required to satisfy each of its three specified criteria. The minimum sample size needed to meet all three criteria (largest of the three calculated sample sizes) was taken as the minimum sample size needed for the development of the prediction model, and we also used the minimum sample size needed to meet only criterion three (precise estimation of the overall risk). So, for a study predicting a binary outcome with 19 candidate predictor parameters, with an outcome proportion of 18.5% and expected Cox-Snell R^2^ value of 0.356, at least 378 participants would be needed to satisfy criteria 1, at least 488 participants for criteria 2 and 232 participants for criteria 3, meaning a minimum overall study sample size of 488 participants (with 91 events, EPP = 91/19 = 4.79) would be needed.

For each study, we calculated the minimum sample size using the outcome proportion from the development data, after any potential data splitting or omission of missing data. Where predictor parameters were not reported or could not be calculated, we use the number of candidate predictors. Where the Cox-Snell R^2^ value was not reported, we used the reported c-statistic to estimate the R^2^ value using the approximation described by Riley et al. [21]. In the absence of a reported R^2^ and c-statistic, we used a conservative value taken as 15% of the maximum R^2^ value to derive the minimum required sample size, as recommended by Riley et al. [14]. We used the ‘pmsampsize’ package in R to calculate the Riley et al. minimum sample size for each study [15]. Using the calculated minimum required sample size, we calculated if studies had ≥ 10 EPP and ≥ 10 EPV.

We described if a study met the calculated minimum sample size to minimise overfitting (criteria 1 and 2) and estimate overall risk precisely (criterion 3), the calculated minimum sample size estimates overall risk precisely (criterion 3) or had an EPV ≥ 10. Differences in the reported sample size of the development data and the calculated sample size, number of events and EPP were described and compared using scatterplots. Depending on the distribution of the data, we log-transformed the sample sizes. The Clopper-Pearson exact method was used to calculate 95% confidence intervals. Data were exported and analysis was conducted in R.

## Results

**Fig. 1:**
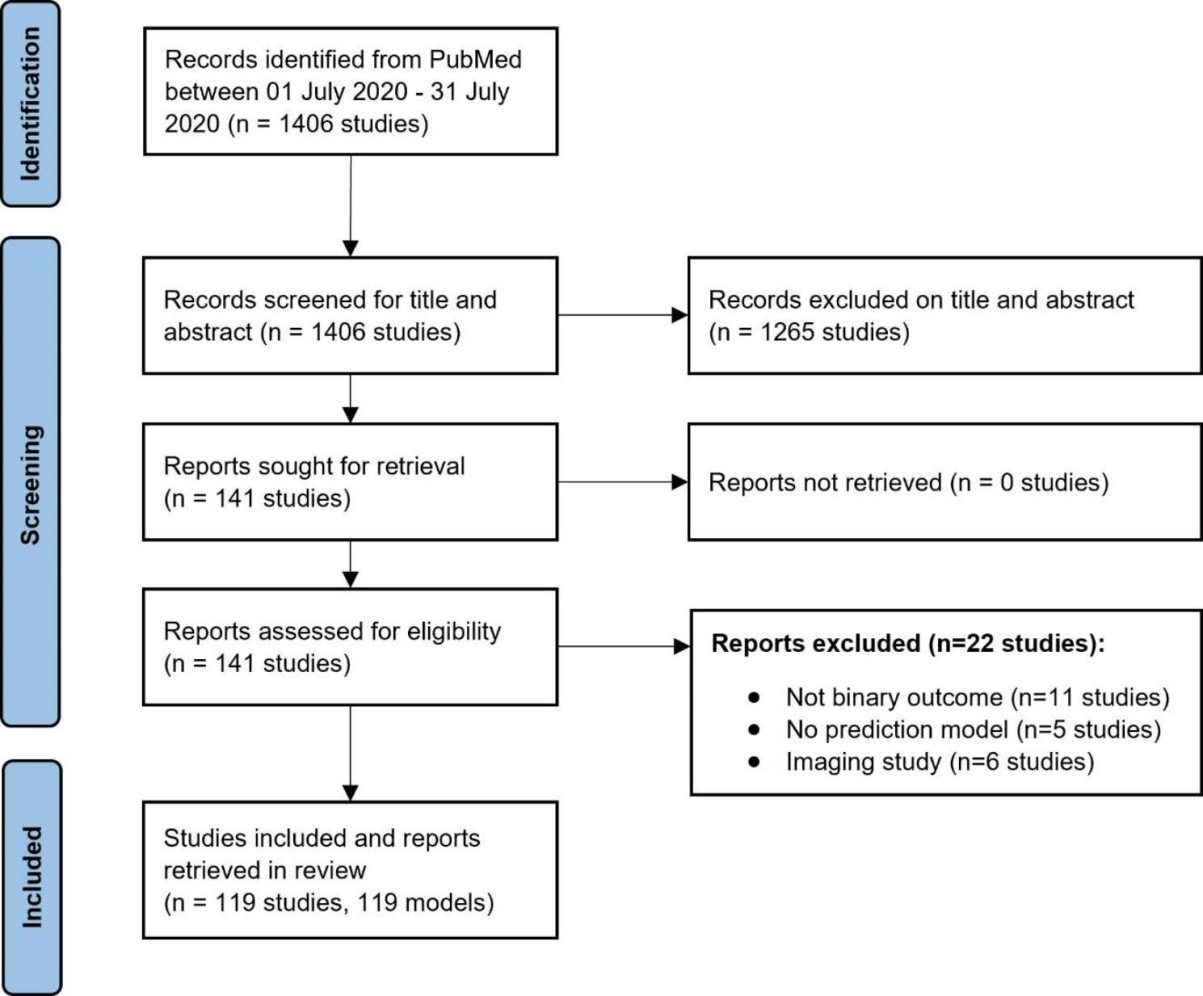
PRISMA flowchart of included studies

The search string identified 1406 studies published and indexed on PubMed between 1 and 2020 and 30 July 2020. The title and abstract screening excluded 1265 studies. The full text of 141 studies were screened, of which 11 studies were not predicting a binary outcome, five studies were not developing a prediction model, and six studies were imaging studies (total 22 studies excluded). Overall, 119 studies were included in the review, and data were extracted on 119 developed models. The flowchart of study selection is presented in Fig. 1.

## Study characteristics

Of the 119 studies, the majority were development only studies (n = 100/119, 84%), i.e., they did not perform an external validation (Table 1). Models were mainly developed using existing data that has already been collected and available for analysis (75/119, 63%) and were most commonly developed in the field of oncology (n = 20/119, 17%). Eighty-eight studies (n = 88/119, 74%) were prognostic and most commonly predicted a health event at a future (short-term) time point (e.g., non-alcoholic fatty liver disease, osteoporosis) (n = 30/88, 34%).

**Table 1:** Study characteristics of included studies (n = 119)

Characteristic	n	%
**Study type**		
Development only	100	84.0%
Development with external validation	19	16.0%
**Data source**		
Existing data	75	63.0%
Prospectively collected cohort	23	19.3%
Registry data	11	9.2%
Case control study	5	4.2%
Cross-sectional	4	3.4%
Randomised controlled trial	1	0.8%
**Clinical specialty**		
Oncology	20	16.8%
Cardiovascular related	19	16.0%
Gastroenterology and hepatology	13	10.9%
Infectious diseases	9	7.6%
Neurology	9	7.6%
Obstetrics and gynaecology	9	7.6%
Endocrinology and metabolic disorders	5	4.2%
Respiratory disorder	5	4.2%
Trauma and Orthopaedics	5	4.2%
Intensive Care Unit	4	3.4%
Radiology	3	2.5%
General population	3	2.5%
Urology	3	2.5%
Haematology	3	2.5%
Surgical patients	2	1.7%
Rheumatology	1	0.8%
Geriatric medicine	1	0.8%
Paediatrics	1	0.8%
Emergency medicine	1	0.8%
Ophthalmology	1	0.8%
Psychiatry	1	0.8%
Not specified	1	0.8%
**Prediction type**		
Prognostic	88	73.9%
Health event*	*30*	*34.1%*
*Mortality*	*19*	*21.6%*
*Complication*	*16*	*18.2%*
Health status**	*14*	*15.9%*
*Treatment related*	*8*	*9.1%*
*Readmission*	*1*	*1.1%*
Diagnostic	31	26.1%
*Disease presence*	*31*	*100.0%*

*Health event = presence of a disease or occurrence of an even at a given time point

**Health status = regression or progression of patient health

### Minimum required sample size

It was possible to calculate the Riley et al. minimum sample size for 94 studies (79%; 95% CI: 71–86%), using the reported number of candidate predictor parameters, outcome proportion and c-statistic. The value of the c-statistic was taken in the following order: bias corrected, split sample or apparent estimate. A bias-corrected c-statistic was used for 47 studies (n = 47/94; 50%, 95% CI: 40–60%), split sample c-statistic was used for 26 studies (n = 26/94; 28%, 95% CI: 19–38%) and apparent c-statistic was used for 21 studies (n = 21/94; 22%, 95% CI: 14–32%).

**Fig. 2:**
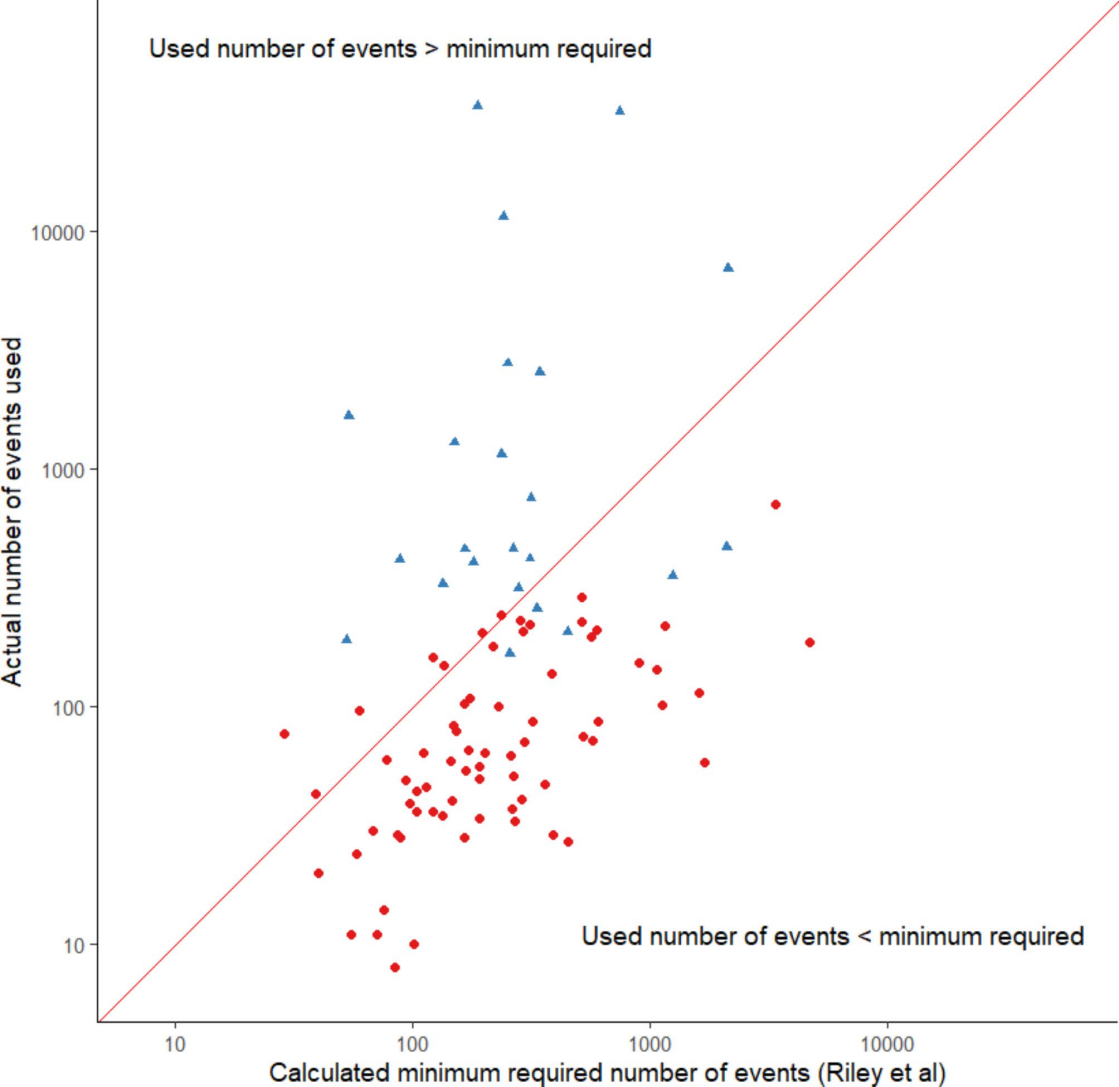
Scatterplot of the actual number of events used to develop the prediction model against the minimum required sample size as calculated by the Riley et al. formulae. Blue triangle = studies where the events per predictor parameter was ≥ 10; red circles = studies where the events per predictor parameter was < 10. The 45-degree reference line indicates where the used sample size was equal to the minimum required sample size

Of the 94 studies a sample size could be calculated for, almost three-quarters did not meet the minimum required sample size to develop their prediction model (n = 69/94; 73%, 95% CI: 63–82%) and a similar number did not meet the ≥ 10 EPV criteria (n = 63/95; 66%, 95% CI: 56–76%) (Fig. 2). Studies that met the minimum required sample size had a median c-statistic of 0.84 (n = 25; IQR: 0.80 to 0.9; range: 0.67 to 0.99) and studies where the minimum sample size was not met had a median c-statistic of 0.83 (n = 69; IQR: 0.75 to 0.9; range: 0.59 to 0.97). The events per predictor parameter was ≥ 10 for five studies that did not meet the minimum required sample size and the events per predictor parameter was < 10 for seven studies that did meet the minimum required sample size. Studies that met the ≥ 10 EPP criteria had a median c-statistic of 0.80 (n = 31; IQR: 0.73 to 0.84; range: 0.59 to 0.98).

Studies were a median of 3.4 events per predictor parameter below the minimum required sample size (median=-3.4 [-8.4 to 0.4]) (Table 2). Studies would be a median of 3 events per predictor parameter below the minimum required sample size (median=-3.0 [-7.7 to 0.4]) if the total available sample size was used to develop the model, i.e., studies did not split their sample or conduct complete case analyses, and this would have resulted in one more study meeting the minimum required sample size (n = 26 studies).

The sample size required to only meet criterion 3 (precise estimation within +/- 0.05 of the average outcome risk) was achieved in a higher number of studies (n = 70/94; 74%; 95% CI: 64–83%) and studies exceeded the minimum requirements by a median of 1.6 events per predictor parameter (median = 1.6 [0 to 6.8]).

**Table 2:** Summary of the calculated minimum required sample size and the difference between observed and calculated estimates where the observed value is: the sample size used to develop the prediction model, or the total available sample size. Values are median [25th and 75th percentiles]

	Total required sample size	Total number of events	Events per predictor parameter
Calculated sample size: Criteria 1 - small overfitting defined by an expected shrinkage of predictor effects by 10% or less (n = 94)	965 [487 to 2268]	188 [101 to 343]	7.8 [4.2 to 14.7]
Calculated sample size: Criteria 2 - small absolute difference of 0.05 in the model’s apparent and adjusted Nagelkerke’s R^2^ value (n = 94)	673 [496 to 1095]	122 [83 to 213]	5.1 [3.9 to 7.6]
Calculated sample size: Criteria 3 - precise estimation (within +/- 0.05) of the average outcome risk (n = 94)	241 [150 to 331]	47 [16 to 108]	1.9 [0.6 to 5.2]
**Minimum required sample size to meet all criteria (n = 94)**	**971 [543 to 2268]**	**211 [114 to 360]**	**9.0 [4.9 to 15.1]**
Difference between minimum required sample size to meet all criteria and sample size that was *used* to develop the model	-387 [-1207 to 49], n = 94	-75 [-234 to 6], n = 94	-3.4 [-8.4 to 0.4], n = 89
Difference between minimum required sample size to meet all criteria and sample size that was *available* to develop the model	-360 [-1207 to 72], n = 94	-63 [-225 to 7], n = 93	-3.0 [-7.7 to 0.4], n = 88
Difference between minimum required sample size to meet criteria 3 and sample size that was *used* to develop the model	262 [-1 to 1165], n = 94	46 [0 to 165], n = 94	1.6 [0 to 6.8], n = 89
Difference between minimum required sample size to meet criteria 3 and sample size that was *available* to develop the model	373 [14 to 1369], n = 94	50 [2 to 210], n = 93	1.9 [0.1 to 9.1], n = 88

### Sample size for model development

Only nine of the 119 studies (8%) reported a sample size calculation or provided a justification of their sample size, of which four studies used the 10 EPV rule of thumb [22–25] and one study used 5 EPV [26]. One study reported ‘we anticipate to evaluate 6/7 independent covariates with high enough (> 2%) prevalence in our sample’[27]. One study only reported the statistical software, alpha value (0.05) and power (80%) [28]. One study reported the sample size calculation in an unmatched case-control study that was used as the data source (‘The formulas of the unmatched case-control study were used to calculate the sample size. The ratio of cases to controls was 1:4. Considering that a larger sample size would yield better performances of the prediction models, 515 patients were randomly selected for inclusion in the study, with 103 patients in the aspiration group and 412 patients in the non-aspiration group’) [29]. One study justified their sample size by reporting ‘BIMS occurred in 2782 patients in our dataset. Simulation studies suggest that this number of events [2782] would allow us to evaluate 100–200 predictor variables without significant risk of statistical overfitting bias [Peduzzi et al, J Clin Epidemiol 1996, 49; Steyerberg et al, J Clin Epidemiol 2011, 64]; we evaluated 41’, referencing research 24 and 9 years old, respectively [30].

The median total sample size and number of events potentially available for model development was 669 (range: 37 to 345,718) and 137 (range: 8 to 48,262), respectively (Table 3).

**Table 3:** Summary of sample size available and used, and the number of candidate predictors and minimum number of candidate predictor parameters for the development of the prediction model

	Number of studies (%)*	Median [p25-p75]	Range
Total available data**	118 (99%)	669 [283–3013]	37 to 345,718
Total available events**	112 (94%)	137 [55–411]	8 to 48,262
Total used data***	119 (100%)	603 [220–2236]	37 to 242,003
Total used events***	97 (82%)	100 [44–243]	8 to 33,784
Total number of candidate predictors	115 (97%)	19 [13–28]	5 to 838
Minimum number of candidate predictor parameters****	110 (92%)	23 [16–31]	7 to 179

*number of studies with sample size information available

**total available refers to the maximum available sample size before any potential data splitting or handling of missing data

***total used refers to the sample size actually used to develop the model, after any potential data splitting or handling of missing data

***minimum number of candidate predictor parameters were calculated by the research team using the reported candidate predictors. This is the minimum number as it does not account for any additional parameters that may be introduced through handling of nonlinear predictors and additional categorisation

### Validation and model performance

Most studies split their available data, where one portion was used for model development and the remaining portion for testing (n = 54/119; 45%, 95% CI: 36–55%), including 11 studies that split their sample in addition to also carrying out bootstrapping (n = 4) or cross validation (n = 6) or both (n = 1) to internally validate their model. Data was split randomly in 39 studies (n = 39/54; 72%, 95% CI: 58–84%), temporally (by time) in 13 studies (n = 13/54; 24%, 95% CI: 13–38%), split by surgeon in one study and the criterion for splitting was unclear in one study. When split, the mean proportion that was allocated to developing the model was 69% (SD: 8.9%).

Of the 39 studies that conducted bootstrapping, the median of the maximum number of bootstrap samples was 1000 (range: 100 to 10,000). For three studies, the number of bootstraps were unclear. Of the 19 studies that conducted cross-validation, nine conducted 10-fold cross validation, three conducted 5-fold cross validation, two conducted leave-on-out cross-validation, and for four studies it was unclear. Eleven studies used the development data to evaluate the performance of their models and for nine studies the method of evaluating model performance was unclear.

Discrimination (measured using the c-statistic) was reported in almost all studies, with at least one estimate (apparent, split sample or bias corrected) reported in 97% of studies (n = 116/119; 95% CI: 93–99%), including 16 studies that also reported an external validation c-statistic estimate (Table 4). The median bias corrected c-statistic was 0.84 (range: 0.59 to 1.00). A calibration plot was only presented in 37% of studies (n = 44/119; 95% CI: 28–46%), of which two-thirds provided a smoothed curve (n = 29/44; 66%; 95% CI: 50–80%). Summary estimates of calibration (slope or calibration in the large) were only reported in 8% of studies (n = 10/119; 95% CI: 4–15%).

**Table 4:** Summary of model performance for the developed model by apparent, split sample, bias-corrected internal validation and external validation estimates

Model performance measure	Number of studies (%, 95% CI)	Median [p25-p75]	Range
**R-squared reported**	**11 (9%, 95% CI: 5–16%)**		
Apparent*	1	0.06 [-]	-
*Split sample*	6	0.31 [0.15–0.34]	0.12 to 0.45
*Bias corrected*	5	0.35 [0.11–0.64]	0.01 to 0.89
External validation*	1	0.88 [-]	-
**C-statistic reported**	**116 (97%, 95% CI: 92–99%)**		
*Apparent*	52	0.84 [0.76–0.91]	0.63 to 0.99
*Split sample*	43	0.81 [0.74–0.88]	0.61 to 1.00
*Bias corrected*	53	0.84 [0.75–0.90]	0.59 to 1.00
*External validation*	16	0.81 [0.73–0.88]	0.67 to 0.96
**Calibration-in-the-large reported**	**10 (8%, 95% CI: 4–15%)**		
*Apparent*	10	0.01 [-0.02–0.18]	-0.26 to 1.82
Split sample*	1	-0.01 [-]	-
*Bias corrected*	0	-	-
*External validation*	0	-	-
**Calibration slope reported**	**9 (8%, 95% CI: 4–14%)**		
*Apparent*	9	0.99 [0.98 to 1.00]	-0.22 to 1.02
Split sample*	1	1.02 [-]	-
*Bias corrected*	0	-	-
*External validation*	0	-	-

*25th, 75th and range not specified as only one model performance estimate was reported

### Reporting standards

Nineteen studies reported using a reporting guideline (n = 19/119; 16%, 10–24%), of which 16 studies used the recommended and applicable reporting guideline for multivariable prediction modelling studies (TRIPOD), but only three reported information on their sample size calculation (reporting using 5, 10 and 10.5 EPV). One study used STROCSS 2021: Strengthening the reporting of cohort, cross-sectional and case-control studies in surgery, one study used The Strengthening the Reporting of Observational Studies in Epidemiology (STROBE) Statement, and one study used the CONSORT 2010 Statement: updated guidelines for reporting parallel group randomised trials.

## Discussion

### Summary of findings

We reviewed the sample size for 119 studies which developed models for predicting a binary outcome using logistic regression. The included studies largely either did not report or mention sample size or did not use a recommended sample size calculation to develop their prediction model. As a result, most studies had insufficient data to reliably develop their models and were thus at risk of overfitting, with 73% not meeting the minimum required sample size for their prediction scenarios. Studies fell short of the minimum required sample size by a median of 75 events per study (EPP: 3.4); a deficit that was exacerbated by use of random split sampling to internally validate models, thereby discarding potential development data. However, even if the total available data was used to develop the model, we found that studies still fell short of the minimum required sample size by a median of 63 events per study (EPP: 3), indicating an issue with defining sample size at the study design stage.

We compared the used and available sample size with the sample size required to only estimate overall risk precisely (criterion 3). Though we found a higher proportion of studies with enough data to meet this criterion (74%), studies only just exceeded the minimum required sample size to meet it.

Where possible, we estimated the number of predictor parameters from the number of candidate predictors considered for the prediction model and how they were handled in studies. We therefore used the minimum number of predictor parameters in our sample size calculations, and so the true deficit in sample size may be higher. In our estimation of predictor parameters, we also included any interactions that were explored and would result in additional terms in the model that would need to be estimated and thus additional terms that would need to inform sample size calculations. We note that the median c-statistic for studies meeting the minimum sample size requirement was higher (0.84), compared to those that did not (0.83) and studies that met the 10EPV rule of thumb (0.80).

### Current literature

Our findings add to current evidence on the limited reporting, planning and methodological conduct of sample size calculations in research on prediction model using logistic regression. Many studies have found a lack of reporting of sample size calculations in prediction model research, whether for development or validation [31, 32]. Further, studies have also found that even when reported, sample size calculations are poorly reported and do not follow recommended guidance[4, 8, 9]. Few studies, however, provide more detail about the quality of sample size calculations and provide any estimation of how close or far studies are from the minimum required sample size for their bespoke prediction model scenario.

Collins et al. reviewed the sample sizes in prediction model studies in prostate cancer and found fewer studies reporting a sample size calculation (three out of 139 studies (2%) compared to nine out of 119 (8%) in the current study) [33]. All three calculations were not following recommended approaches, with two studies using ≥ 10EPV and ≥ 20EPV rules of thumb and one study basing it on a comparative power calculation. Collins et al. also found a low number of studies satisfying the Riley et al. minimum sample size requirements, but the overall proportion was higher than the findings in the present review (51% vs. 23%, respectively).

The Collins et al. prostate cancer review uses a sample of studies published from 1994 up to 30 June 2019 and is limited because the Riley et al. sample size formulae were not published till March 2019 and so would not have been available for most (if not all) of the included studies, which most likely will have commenced before March 2019. Our study builds on this review by taking a prospective view after the Riley et al. sample size formulae became available and allowing a longer time period to elapse from publication of the Riley et al. sample size formulae and publication of the studies in the review, increasing the probability of exposure to the more formal calculations. We also take a broader look of current practice around sample size in prediction model research by not limiting our search to any clinical specialty.

### Strengths and limitations

At the time the search was run, we took a contemporary sample of papers from 2020 but now the search results are almost 3 years old. This is in part due to delays related to the disruptive effects of the COVID-19 pandemic. However, given the slow pace of prediction model methodological research and advancement, and owing to the fact the formal sample size guidance was published in 2019, we believe that our findings are relevant and still reflective of current practice in prediction model research.

We also acknowledge that the Riley et al. formulae for sample size calculation were published in March 2019 and our search results were from July 2020. Arguably, more time may need to have elapsed for study teams to be aware of and use the new guidance at the time of commencing their prediction model studies. However, our study does provide recommendations with respect to how future researchers can calculate the minimum sample size requirement within methodological recommendations in prediction model research. We also provide additional information (such as total available and used sample size) and estimation of the amount by which studies fail to meet recommended minimum sample size requirements. We also provide the benchmark for other studies evaluating the presence and reporting quality of sample size calculation and estimating deficit of sample sizes that are used to develop prediction models.

### Future research and recommendations

Sample size is one of the minimum reporting recommendations in the TRIPOD reporting guideline and signalling question for PROBAST the risk of bias tool for prediction model studies [1, 2, 34, 35]. We recommend study teams developing or validating a prediction model, at a minimum, fully and transparently report their sample size calculation or justification irrespective of the method used. This will allow better evaluation of studies and models, whether it be at peer review for publication or for evidence synthesis or national guideline development.

We do not recommend that study teams use the 10 EPV rule of thumb or using any other arbitrary EPV value to inform sample size calculation, and encourage study teams to use more formal sample size guidance that is available for prediction model development and validation [12, 13, 36–38]. We highlight that these formulae are not limited to regression modelling approaches but are also applicable to non-regression modelling approaches, such as machine learning, as they are based on the outcome that is to be predicted (e.g., continuous, binary, time-to-event, multinomial).

We also recommend assessing and reporting model stability (stability in the predicted risks) using instability plots as detailed by Riley et al. [3], especially if too small sample sizes have been used and minimum sample size requirements have not been met. In the instance of potentially small sample sizes, it is more important to demonstrate that predictions from the developed model are stable, as unstable and unreliable predictions could lead to patient harm.

We also encourage future reviews of prediction models to include detailed evaluations of the reporting and conduct of sample calculation and where possible, to summarise the difference between the minimum required sample size and the sample size that was used. This will provide ongoing evidence of adherence to sample size reporting guidance and the uptake of more formal sample size guidance.

## Conclusion

Sample size calculation and justification is rarely reported in studies developing a prediction model for a binary outcome using logistic regression and studies often do not use enough data to meet minimum sample size requirements for their prediction model scenario. Models developed using insufficient data will lead to model instability and unreliable predictions, that if used to guide clinical decision making have the potential to cause harm. With formal sample size and reporting guidance available, we strongly encourage researchers to fully and transparently perform and report their sample size calculations, so they meet minimum sample size and reporting requirements for their studies.

### Electronic supplementary material

Below is the link to the electronic supplementary material.

Supplementary Material 1

## Data Availability

The datasets generated and/or analysed during the current study are available in the Open Science Framework repository (osf.io/qydmk).
