# Supplementary material for: Sample size requirements are not being considered in studies developing prediction models for binary outcomes: a systematic review
Source: BMC Med Res Methodol. 2023 Aug 19;23:188. doi: 10.1186/s12874-023-02008-1 (PMC10439652; doi:10.1186/s12874-023-02008-1)
Supplement: Supplementary file 1 — Supplementary Material 1 [file 12874_2023_2008_MOESM1_ESM.docx]

**Supplementary information**

**Supplementary Box 1: Search string**

| **The final PubMed search strategy is**:  ('prediction'[tiab] OR 'predictive'[tiab] OR 'risk' [tiab] OR 'prognostic'[tiab] OR 'diagnostic'[tiab] 'discrimination'[tiab] OR 'calibration'[tiab] OR 'area under the curve'[tiab] OR 'auc'[tiab] OR 'c-statistic'[tiab] OR 'ROC'[tiab] OR 'area under ROC curve '[tiab] OR 'auROC[tiab] OR 'area under the receiver operating characteristic curve'[tiab] OR 'area under receiver operating characteristic curve'[tiab]) AND ('model'[tiab] OR 'equation'[tiab] OR calculator'[tiab] OR 'assessment'[tiab] OR 'decision making'[tiab] OR 'rule'[tiab] OR 'score'[tiab] OR 'risk model'[tiab] OR 'statistical model'[tiab] OR 'multivariable'[tiab] OR 'statistical regression'[tiab] OR 'risk score'[tiab] OR 'logistic'[tiab] OR 'cox'[tiab] OR 'survival'[tiab] OR 'regression'[tiab]) NOT ('Radiomics'[tiab] OR 'miRNAs'[tiab] OR 'mRNA'[tiab] OR 'lncRNAs'[tiab] OR 'gene'[tiab] OR 'radio'[tiab] OR 'cell'[tiab] OR 'systematic review'[tiab] OR review[Publication Type] OR Bibliography[Publication Type] OR Editorial[Publication Type] OR Letter[Publication Type] OR Meta-analysis[Publication Type] OR News[Publication Type])  **Run on 03/08/2020** |
| --- |
